# Pollutant-induced cell death and reactive oxygen species accumulation in the aerial roots of Chinese banyan (*Ficus microcarpa*)

**DOI:** 10.1038/srep36276

**Published:** 2016-11-02

**Authors:** Nan Liu, Ce Cao, Zhongyu Sun, Zhifang Lin, Rufang Deng

**Affiliations:** 1 Key Laboratory of Vegetation Restoration and Management of Degraded Ecosystems, South China Botanical Garden, Chinese Academy of Sciences, Guangzhou, 510650 China; 2 University of the Chinese Academy of Sciences, Beijing, 100049 China; 3 Guangdong Open Laboratory of Geospatial Information Technology and Application, Guangzhou Institute of Geography, Guangzhou, 510070 China

## Abstract

Industrial pollutants induce the production of toxic reactive oxygen species (ROS) such as O_2_^.−^, H_2_O_2_, and ^·^OH in plants, but they have not been well quantified or localized in tissues and cells. This study evaluated the pollutant- (HSO_3_^−^, NH_4_NO_3_, Al^3+^, Zn^2+^, and Fe^2+^) induced toxic effects of ROS on the aerial roots of Chinese banyan (*Ficus microcarpa*). Root cell viability was greatly reduced by treatment with 20 mM NaHSO_3_, 20 mM NH_4_NO_3_, 0.2 mM AlCl_3_, 0.2 mM ZnSO_4_, or 0.2 mM FeSO_4_. Biochemical assay and histochemical localization showed that O_2_^.−^ accumulated in roots in response to pollutants, except that the staining of O_2_^.−^ under NaHSO_3_ treatment was not detective. Cytochemical localization further indicated that the generated O_2_^.−^ was present mainly in the root cortex, and pith cells, especially in NH_4_NO_3_- and FeSO_4_-treated roots. The pollutants also caused greatly accumulated H_2_O_2_ and ^·^OH in aerial roots, which finally resulted in lipid peroxidation as indicated by increased malondialdehyde contents. We conclude that the *F. microcarpa* aerial roots are sensitive to pollutant-induced ROS and that the histochemical localization of O_2_^.−^ via nitrotetrazolium blue chloride staining is not effective for detecting the effects of HSO_3_^−^ treatment because of the treatment’s bleaching effect.

China is experiencing serious pollution problems caused by petrochemical smelting, mining, manufacturing, and other activities associated with rapid industrialization. In 2015, China emitted an estimated 18.59 million tons of sulfur dioxide (SO_2_), and 18.51 million tons of nitrogen oxides (NO_x_), and had critical levels of soil pollution by heavy metal^1^. Industrial pollutants, such as SO_2_, NO_x_, NH_3_, and metal ions, are thought to directly or indirectly threaten the health of plants; the symptoms include damaged chloroplast ultrastructure^2^, reduced cell viability^3^,^4^, reduced water-use efficiency^5^, and increased carbon construction costs^6^. Specifically, atmospheric SO_2_ can easily penetrate membranes and convert into bisulfite and sulfite ions in cells^7^,^8^. By opening S-S bridges (sulfitolysis), sulfite can inactivate the proteins in the thioredoxin system and thereby change redox status, light-dark regulation, and chloroplast metabolism^9^,^10^. Atmospheric deposition of nitrogen can directly affect the plant nutrient uptake, growth, and metabolism of plants^11^. The assimilation of NH_4_^+^ can cause cellular acidosis, which alters acid-base regulation in plant cells^12^. Some metals such as aluminium (Al) are redox-inactive and lack metabolic function in plants. Aluminium ion (Al^3+^) or its hydrated form AlCl(H_2_O)_6_^3+^ in acidic tropical soil is toxic to plants causing damage to the cell wall, cytosol, and root cytoskeleton^13^,^14^. Unlike Al, redox-active metals like Zn and Fe are involved in plant metabolism^15^. High levels of Zn can compete with iron, leading to decreased metabolisms in plants^16^. Although plants require Fe, high levels of Fe in soil may cause deficiencies of other nutrients, including P, K, Ca, Mg, and Zn^17^.

Some of the damage caused by industrial pollution to trees results from the induction of oxidative processes that reduce peroxidic bonds and that consequently catalyse the production of reactive oxygen species (ROS), such as superoxide (O_2_^·−^), hydrogen peroxide (H_2_O_2_), and hydroxyl radical (^·^OH)^2^,^3^,^18^. SO_2_ phytotoxicity is mainly attributed to the production of intracellular O_2_^·−^, and its detoxification is primarily dependent on the oxidative conversion of SO_3_^2−^ and HSO_3_^−^ into non-harmful sulfate (SO_4_^2−^)^19^ Oxides of nitrogen (NO and NO_2_) also cause oxidative stress to plants. NO can rapidly react with O_2_^.−^ to form ONOO^−^, which may transform to ^·^OH, the most reactive and toxic ROS^20^,^21^. Moreover, in the presence of nitrate (NO_3_^−^) assimilation, ^·^OH can be generated and cause free radical-induced injury^22^. Zn and Fe are redox-active metals, and redox cycling catalyses the production of ROS through the Fenton reaction or the peroxidase-catalysed reaction in the presence of O_2_ and NADH^23^. As a redox-inactive metal, Al cannot directly participate in biological redox reactions with oxygen, but it can inhibit antioxidant enzymes, causing the accumulation of ROS in cells. ROS can cause cell death and organ senescence, because they readily participate in chain reactions between free radicals and membrane lipids and proteins, resulting in the breakdown of membranes, disturbance of mitosis, inhibition of DNA synthesis, and inactivation of enzymes^4^,^20^,^24^.

The deposition of atmospheric sulfur, nitrogen, and industrial dust containing metals has caused the decline of indigenous tree species in South China, but the mechanisms are incompletely understood^5^,^6^,^25^. Our previous studies indicated that different forms of pollutants, alone or in combinations, are involved in accelerating oxidative process, causing decreased rates of electron transport and damaged membrane systems in leaf cells^2^,^5^,^6^. The toxic effects of ROS caused by various pollutants on aerial roots, however, have been rarely investigated or compared^18^. Aerial roots directly contact air and soil pollutants, and their growth was found to be restricted in industrially polluted regions in subtropical China^26^. In the current study, we evaluated the oxidative stress induced by various industrial pollutants in the aerial roots of Chinese banyan. Chinese banyan is a common landscape tree with a unique aerial root system that grows downward along the trunk to the soil^27^. We also compare methods for quantifying ROS.

## Methods

### Plant material and pollutants

Chinese banyan, *Ficus microcarpa* Linn. f. (Moraceae), is a native evergreen tree that is used for urban greening in South China^27^. In April 2015, newly sprouted aerial roots were removed from 15 mature trees growing in the South China Botanical Garden, Guangzhou, China. Each aerial root segment was 5 cm long had a root tip on one end. The root segments were quickly transferred to the laboratory and rinsed with distilled water and then wiped dry.

The aerial root samples (6–8 for per tree from 15 trees) were vacuum-infiltrated for 30 min with distilled water (control, pH 6.09), 20 mM NaHSO_3_ (pH 3.08), 20 mM NH_4_NO_3_ (pH 4.86), 0.2 mM AlCl_3_ (pH 4.07), 0.2 mM ZnSO_4_ (pH 5.48), or 0.2 mM FeSO_4_ (pH 4.47). Vacuum infiltration was used in order to decrease the differences in the penetration rates of the different ions into the root segments and shorten the treatment period. We referred the atmospheric sulfur and nitrogen, and surface soil metal concentrations in industrially polluted site in South China as background information^2^,^5^,^18^,^26^,^28^. Based on these reported and our preliminary data, we treated our aerial root samples by designated pollutant concentrations as mentioned above. During treatment, the root samples were kept in an incubator (10 h light and 14 h dark) at 25 °C. A subsample of each root sample (a 3-cm length from the root tip) was then used to determine aerial root viability, the histochemical localization of ROS, and ROS content as described in the following sections.

### Aerial root viability

Aerial root viability was determined by Evans blue staining^29^. The 3-cm-long subsamples (five per treatment) were immersed in a 0.25% solution of Evans blue (E2129, Sigma); after 12 h, the subsamples were washed with distilled water to remove the Evans blue solution from the root surface. The dyed root samples were photographed with a digital camera (DSC-F717, Sony, Japan) and then chopped into small pieces and placed in a 1% sodium dodecyl sulfate (SDS) solution for 24 h to completely extract the blue stain. The blue extract, which represented dead cells, was quantified with a spectrophotometer (Lambda 650, Perkin-Elmer, USA) at 600 nm.

### Histochemical and cytochemical localization of O_2_^.−^

O_2_^.−^ was localized by staining with nitrotetrazolium blue chloride (NBT, N6876, Sigma)^18^. The 3-cm-long subsamples (five per treatment) were immersed in HEPES-NaOH buffer (pH 7.6) containing 0.5 mg of NBT/ml and 10 mM NaN_3_. The subsamples were vacuum infiltrated in this NBT solution for 30 min and were then held at room temperature until the blue colour (NBT-O_2_^.−^) became visible. The NBT-stained roots were photographed with a digital camera (DSC-F717, Sony, Japan) before semi-thin transverse sections (8 μm thick) were prepared. Semi-thin section was conducted by fixing aerial root samples in 0.1 M phosphate buffer (pH 7.2) containing 2% glutaraldehyde and 2.5% Paraformaldehyde. After 6 times wash with 0.1 M phosphate buffer, they were dehydrated by alcohol steeply and eddied in flat molds using EPON812 resin. Sctions (2 μm) were cut by ultramicrotome (Leica, UC6, Germany). The sections were observed and photographed with a light microscope (AX70, Olympus, Japan) and a digital camera (DP50, Olympus, Japan).

### Histochemical localization of H_2_O_2_

H_2_O_2_ was localized by staining with 3,3′,5,5′-Tetramerthyl benzidine dihydrochloride hydrate (TMB, V900355, Sigma)^30^. The 3-cm-long subsamples (five per treatment) were immersed in 10 mM sodium-citrate buffer (pH 4.0) containing 1 mM TMB at room temperature until the TMB-H_2_O_2_ formazan became visible. The stained roots were then photographed with a digital camera (DSC-F717, Sony, Japan).

### ^·^OH and H_2_O_2_ quantification

^·^OH was quantified using terephthalic acid (TPA) as a hydroxyl radical dosimeter as described in previous studies^21^,^29^. The 3-cm-long subsamples (five per treatment) were homogenized in phosphate buffer (50 mM, pH 7.0), and the supernatant was collected after centrifugation at 10000 *g* for 10 min at 4 °C. The 0.2-ml extracts were incubated in a 2-ml solution containing 0.2 ml of 50 μM TPA and 1.6 ml of phosphate buffer (50 mM, pH 7.0). After incubation for 10 min, the fluorescence emission spectra from 350 to 550 nm of monohydroxy terephthalate (TPA-^·^OH) was recorded with a fluorescence spectrophotometer (LS 55, Perkin-Elmer, USA) with an excitation wavelength of 326 nm.

H_2_O_2_ was detected using a fluorescence spectrophotometer (LS 55, Perkin-Elmer, USA) as previously described^18^,^31^. The 3-cm-long subsamples (five per treatment) were homogenized in phosphate buffer (20 mM, pH 6.0). After the homogenate was centrifuged at 10000 *g* for 10 min at 4 °C, 5 ml of the supernatant was collected. The 3-ml reaction mixture also included 0.2 ml of root extract, 5 μM scopoletin (S2500, Sigma), and 3 μg ml^−1^ horseradish peroxidase. The fluorescence emission spectra were recorded from 400 to 550 nm with an excitation wavelength of 346 nm.

### Detection of O_2_^.−^ accumulation

Root sample extracts were obtained after homogenization in phosphate buffer (20 mM, pH 6.0). The 0.2-ml extracts were then incubated for 5 h in the dark in 2 ml of phosphate buffer (20 mM, pH 6.0) containing 0.5 mM Na, 39- [1-[(phenylamino)-carbonyl]-3,4-tetrazolium]-bis(4-methoxy-6-nitro) benzenesulfonic acid hydrate (XTT, X4626, Sigma). Formation of XTT-O_2_^.−^-formazan was detected using a UV spectrophotometer (Lambda 650, Perkin-Elmer, USA) at 470 nm^18^,^31^.

### Malondialdehyde (MDA) quantification

Root samples were homogenized with 0.5% (w/v) thiobarbituric acid in 20% (w/v) trichloroacetic acid. The mixture was incubated at boiling water for 30 min and then quickly cooled in a refrigerator. After centrifugation at 1800 g for 10 min, the supernatant was used for MDA determination using a UV spectrophotometer (Lambda 650, Perkin-Elmer, USA)^32^.

### Data analysis

Results are shown as means ± standard deviations (SDs). One-way analyses of variance (ANOVAs) were used to determine the effects of treatment on Evans blue staining, XTT-O_2_^.−^ formation and MDA quantification. When effects were significant, means were compared with the Tukey’s test. All statistical analyses were performed by SPSS 19.0 (SPSS, Inc., USA). Differences were considered significant at P < 0.05.

## Results

### Aerial root viability

**Figure 1:**
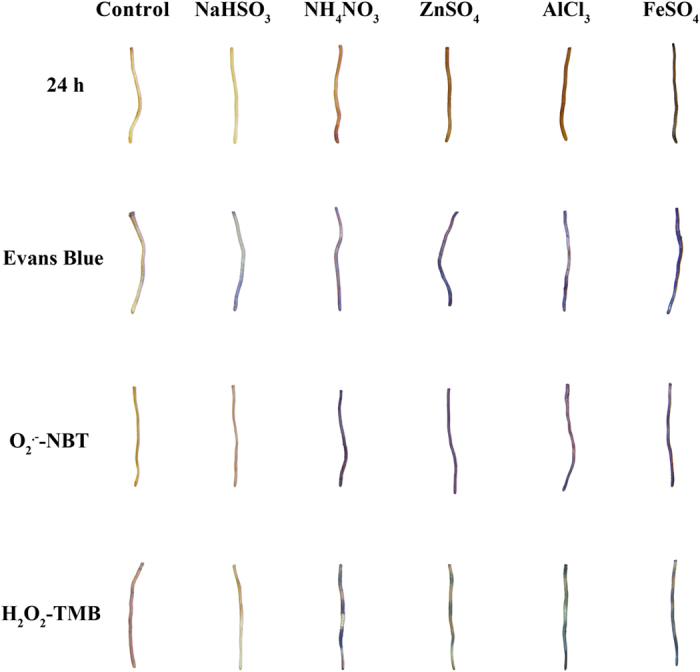
Surface colour (first row), cell viability (second row), and histochemical localization of O_2_^.−^ (third row) and H_2_O_2_ (last row) in aerial roots of *F. microcarpa* treated with purified water (Control), 20 mM NaHSO_3_, 20 mM NH_4_NO_3_, 0.2 mM ZnSO_4_, 0.2 mM AlCl_3_, or 0.2 mM FeSO_4_. The roots were photographed after 24 h of treatment. Cell viability is indicated by the Evans blue staining. O_2_^.−^ and H_2_O_2_ accumulations are indicated by the formation of NBT-O_2_^.−^ and TMB-H_2_O_2_.

**Figure 2:**
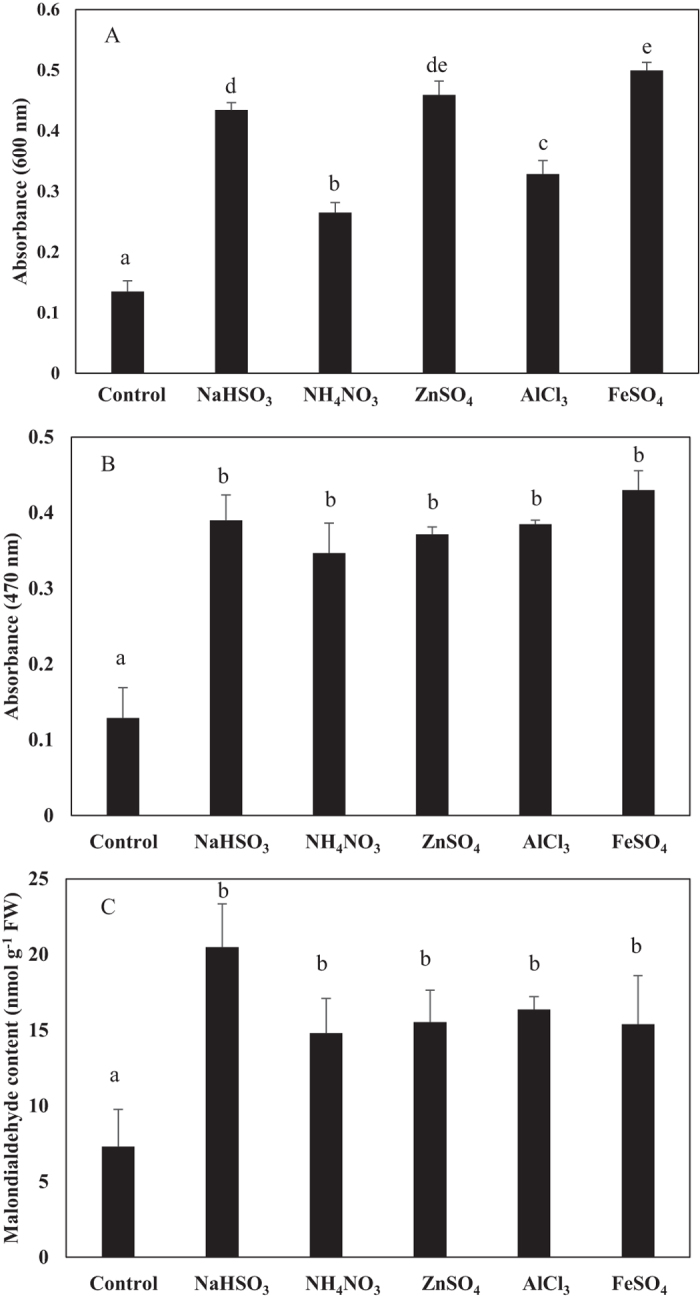
Cell viability indicated by absorbance of the Evans blue extract at 600 nm (**A**), O_2_^**.−**^ accumulation as indicated by absorbance of XTT-O_2_^.−^-formazan at 470 nm (**B**), and levels of lipid peroxidation as indicated by malondialdehyde content (**C**) in aerial roots of *F. microcarpa* treated with purified water (Control), 20 mM NaHSO_3_, 20 mM NH_4_NO_3_, 0.2 mM ZnSO_4_, 0.2 mM AlCl_3_, or 0.2 mM FeSO_4_. Values are means + SD; n = 6. Means with different letters are significantly different at P < 0.05.

The surface colour of roots incubated in NH_4_NO_3_, ZnSO_4_, AlCl_3_, or FeSO_4_ became darker relative to the control, while the surface colour of roots incubated in NaHSO_3_ became lighter (Fig. 1). As indicated by Evans blue staining, the pollutants reduced cell viability (Fig. 1). The blue staining mainly occurred in the root tips after treatment with NaHSO_3_ but occurred throughout the 3-cm-long subsample following treatment with NH_4_NO_3_, ZnSO_4_, AlCl_3_, or FeSO_4_. The absorbance of the blue extract (600 nm) confirmed that all of the pollutants significantly reduced the viability of the aerial roots (P < 0.05, Fig. 2A). Moreover, the viability was lower following NaHSO_3_, ZnSO_4_, and FeSO_4_ treatment than following NH_4_NO_3_ or AlCl_3_ treatment.

### Histochemical and cytochemical localization of O_2_^.−^

**Figure 3:**
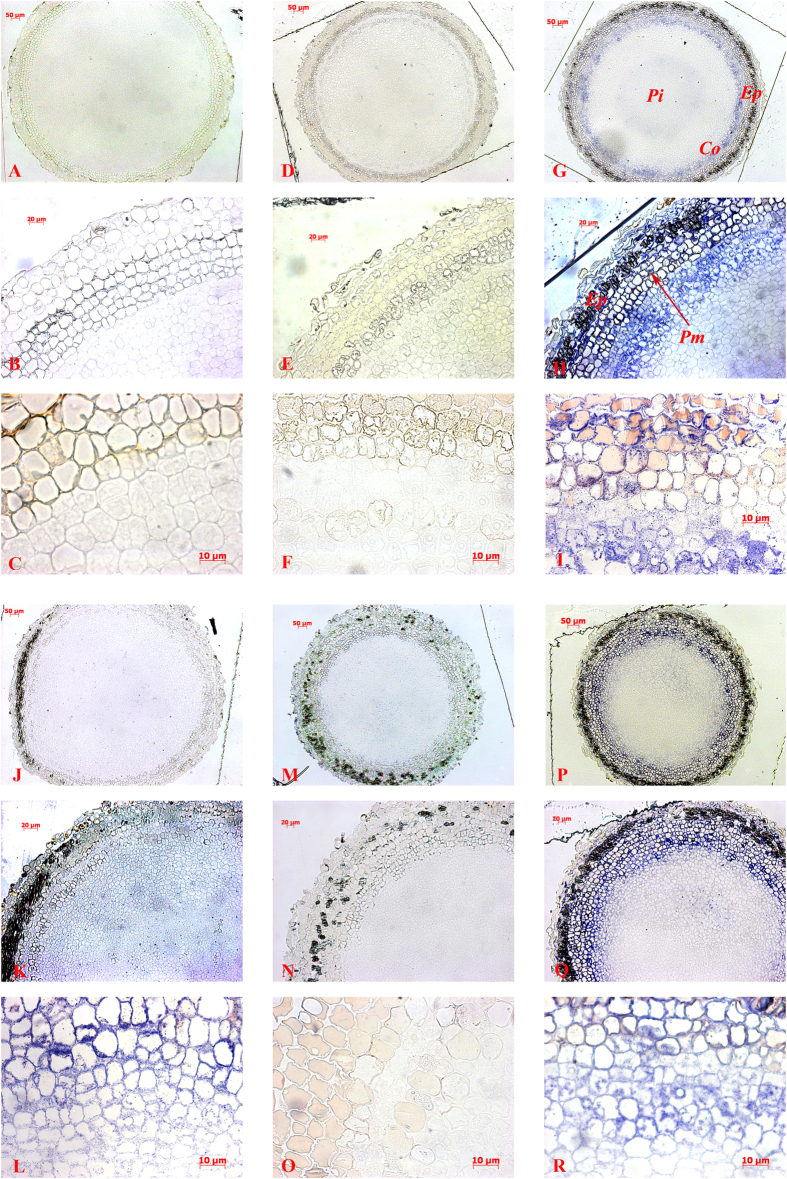
Cytochemical localization of O_2_^.−^ in aerial root cells (cross section behind root tips) of *F. microcarpa* treated with purified water (**A–C**), 20 mM NaHSO_3_ (**D–F**), 20 mM NH_4_NO_3_ (**G–I**), 0.2 mM ZnSO_4_ (**J–L**), 0.2 mM AlCl_3_ (**M–O**), or 0.2 mM FeSO_4_ (**P–R**). (**C,F,I,L,O,R**) are enlarged pictures of corresponding (**B,E,H,K,N,Q**) by the order of 100 times, respectively. Co: cortex; Ep: epidermis; Pi: pith; Pm: plasma membrane.

When dyed with NBT, root segments treated with NH_4_NO_3_, ZnSO_4_, AlCl_3_, or FeSO_4_ but not with NaHSO_3_ became blue, indicating the presence of O_2_^.−^ (Fig. 1). The blue was most intense in roots treated with NH_4_NO_3_ and FeSO_4_. Semi-thin transverse sections indicated that large quantities of blue formazan (NBT-O_2_^.−^) accumulated in the root tips following treatment with NH_4_NO_3_ (Fig. 3G–I) and the metal pollutants (Fig. 3J–R) and that most of the O_2_^.−^ was in the root cortex and pith cells. In contrast, cross sections of control root tips or those treated with NaHSO_3_ were not blue (Fig. 3A–F). In agreement with the histochemical observations, the cytochemical localizations of O_2_^.−^ indicated that accumulation of O_2_^.−^ was greater following treatment with NH_4_NO_3_ and FeSO_4_ than in the control. Although large quantities of ROS were generated in the root tips following treatment with the pollutants for 24 h, pollutant-induced damage to cell structure was not evident in the enlarged microscopic pictures (Fig. 3C,I,L,O,R).

### Histochemical localization and quantification of O_2_^.−^ and ^·^OH

As indicated by XTT-O_2_^.−^-formazan absorbance at 470 nm, O_2_^.−^ accumulation in aerial root cells was significantly higher in all of the pollutant treatments than in the control (P < 0.05, Fig. 2B). The significantly elevated O_2_^.−^ accumulation induced by the pollutants is consistent with the NBT staining of root cross sections (Fig. 1).

**Figure 4:**
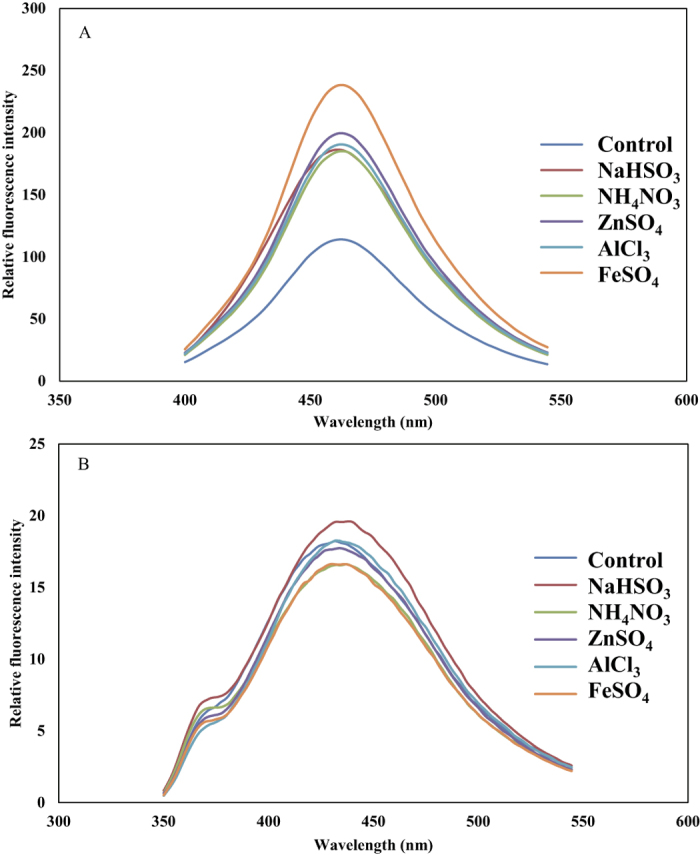
^·^OH accumulation as indicated by the fluorescence intensity of TPA-^·^OH formazan at 463 nm (**A**), and H_2_O_2_ accumulation as indicated by the fluorescence intensity of H_2_O_2_-scopoletin formazan at 433 nm (**B**) in aerial roots of *F. microcarpa* treated with purified water (Control), 20 mM NaHSO_3_, 20 mM NH_4_NO_3_, 0.2 mM ZnSO_4_, 0.2 mM AlCl_3_, or 0.2 mM FeSO_4_. Each curve is the average of 5–6 replicates.

To assess the accumulation of ^·^OH, fluorescence spectra were detected by adding TPA to the root extracts. The TPA-^·^OH fluorescent emission curves peaked at 463 nm, and the intensities were much higher for roots treated with pollutants than for control roots (Fig. 4A). The peak of the relative fluorescent values of TPA-^·^OH was higher for FeSO_4_ than for the other pollutants.

### Quantification of H_2_O_2_

The accumulation of H_2_O_2_ was assessed using fluorescence spectra by adding scopoletin to the root extracts. H_2_O_2_ accumulation (based on relative fluorescence intensity at 433 nm) in aerial root segments did not substantially differ between the control and the other treatments (Fig. 4B). Different from fluorescent assays, the histochemical staining of TMB-H_2_O_2_ showed that root segments treated with NH_4_NO_3_, ZnSO_4_, AlCl_3_, or FeSO_4_ was obvious, indicating the presence of H_2_O_2_. By contrary, the staining of TMB-H_2_O_2_ on NaHSO_3_ treated root samples was not detected (Fig. 1).

### Quantification of MDA

The MDA contents of pollutant-treated aerial root samples were mostly higher than that of controls. The significantly increased MDA levels were detected in all pollutant treated root samples, indicating higher oxidative damage and lipid peroxidation (Fig. 2C).

## Discussion

In this study, aerial roots of Chinese Banyan obviously suffered from treatment with pollutants as indicated by darker root surfaces (except in the case of NaHSO_3_), dehydration symptoms (especially in the case of FeSO_4_), and accumulation of ROS. Bisulfite (HSO_3_^−^) is the byproduct of SO_2_ in cells, and the derivative is directly and indirectly toxic to plant tissues^2^. SO_2_ and its derivate HSO_3_^−^ harm leaves by generating excessive quantities of ROS, resulting in the bleaching of photosynthetic pigments^33^,^34^. Our study found, for the first time to our knowledge, that aerial root systems were also harmed by bleaching caused by HSO_3_^−^, the cell death caused by NaHSO_3_ was confirmed by Evans blue staining (Fig. 1). Similarly, Evans blue staining in this study indicated that the viability of aerial root cells was decreased by NH_4_NO_3_, ZnSO_4_, AlCl_3_, and FeSO_4_ (Fig. 1). The decrease of aerial root cell viability was mainly caused by the decrease of cell pH, imbalance of mineral assimilation, as well as injuries in cell wall, plasma membrane, and signal transduction pathways^11^,^12^,^13^,^14^,^15^,^16^.

Under biotic and abiotic stress, plant cells produce ROS in several subcellular compartments^35^. As revealed by previous studies, redox-active metals (e.g., Fe^2+^ and Zn^2+^) as well as redox-inactive metals (e.g., Al^3+^) may induce the activity of plasma membrane-localized NADPH oxidase, which transfers electrons from cytosolic NADPH to O_2_ and subsequently forms O_2_^.−^36,37. In our study, the deep-blue staining of NBT-O_2_^.−^-formazan in the aerial roots that were treated with metal pollutants was documented by histochemical staining and by cytochemical observation of micrographs; our cytochemical observations were consistent with previous reports that NBT-O_2_^.−^ is mainly found in cells^18^. Thus, we infer that the increased absorbance by XTT-O_2_^.−^ at 470 nm and the formation of NBT-O_2_^.−^ can be attributed to the activation of NADPH oxidase by metal ions in aerial roots. High concentrations of NaHSO_3_ and NH_4_^+^ have been reported to damage cells because HSO_3_^−^ detoxification and NO_3_^−^ assimilation cause the generation of free radicals^2^,^12^,^20^. In accordance with these studies, our results showed that all pollutants caused massive accumulations of O_2_^.−^ in cells, as indicated by biochemical assay (Fig. 2B) and by histochemical staining (Fig. 3). The exception was that only low levels of O_2_^.−^ were detected in NaHSO_3_ treated root segments: even though XTT-O_2_^.−^ absorbance was high, NBT-O_2_^.−^-formazan was almost undetectable by histochemical and cytochemical observation (Fig. 3D–F). NaHSO_3_ is usually used as an additive bleaching agent. Therefore, we suspect that the bleaching caused by HSO_3_^−^ may result in the failure of NBT staining and that NBT-O_2_^.−^ staining is not suitable for O_2_^.−^ detection in SO_2_- or HSO_3_^−^-treated tissues.

In our study, H_2_O_2_ accumulation was not detected in pollutant-treated tissues by the fluorometric scopoletin oxidation assay (Fig. 4B). The histochemical staining, however, clearly indicated the production of H_2_O_2_ in pollutant-treated aerial root samples. Here, we infer that H_2_O_2_ detective method by fluorescence intensity of H_2_O_2_-formazan may not always be effective, because the peroxide activity might be enhanced during the preparation of root extract, causing more consumption of H_2_O_2_ and reduced fluorescence intensity of H_2_O_2_-fomazan. This result was also in agree with previous study that H_2_O_2_ is a versatile member of ROS network and that H_2_O_2_ increased in plant tissues under Al stress^38^,^39^,^40^.

^·^OH is among the most toxic of the ROS because of its capacity to initiate radical chain reactions that result in irreversible chemical modifications of various cellular components^41^. Because different pollutants (SO_2_, NH_4_NO_3_, and metal ions) are all involved in the accumulation of ROS within plant cells^2^,^20^,^29^, the induced oxidative processes finally break the free radical chains of membrane lipids, causing membrane decomposition (increased MDA content, Fig. 2C) and cell death (decreased cell viability, Fig. 2A). In our study, TPA-^·^OH fluorescence was greatly increased by all five pollutants, indicating that these pollutants increased ^·^OH accumulation in aerial root tissues (Fig. 4A). Because Fe^2+^ is involved in the Fenton reaction, FeSO_4_ treatment greatly increased ^·^OH concentrations in aerial root tissues. NH_4_NO_3_ treatment also greatly increased ^·^OH accumulation in aerial root tissue, which is consistent with previous findings that nitrate assimilation directly interferes with free radical metabolism and causes free radical-induced injury^20^.

Overall, the pollutant treatments in the current study caused ROS accumulation and profound oxidative damage, and finally cell death in aerial root tissues. Because O_2_^.−^ is the initial ROS generated during O_2_ metabolism in plant tissue, quantification of O_2_^.−^ is vital for assessing ROS damage in plant tissues subjected to various stresses. In our study, we used both XTT and NBT to detect the accumulation of O_2_^.−^. XTT is more sensitive than NBT, and XTT-O_2_^.−^ can be quantitatively detected using spectrochemical methods. NBT staining may be suitable for the qualitative assessment of O_2_^.−^ accumulation in plant tissues that have been subjected to most stresses but not to NaHSO_3_. The bleaching effect of HSO_3_^−^ reduced the effectiveness of NBT staining in plant tissues. This study also showed that the aerial roots of *Ficus microcarpa* are sensitive to various pollutants and that aerial roots may be good indicators of pollutants in industrially polluted regions.

## Additional Information

**How to cite this article**: Liu, N. *et al*. Pollutant-induced cell death and reactive oxygen species accumulation in the aerial roots of Chinese banyan (*Ficus microcarpa*). *Sci. Rep*. **6**, 36276; doi: 10.1038/srep36276 (2016).

**Publisher’s note:** Springer Nature remains neutral with regard to jurisdictional claims in published maps and institutional affiliations.
